# Bradyarrhythmias and Brugada syndrome: clinical features, long-term outcome and prognostic implications*—the BruBra study*

**DOI:** 10.1093/europace/euag131

**Published:** 2026-05-27

**Authors:** Tardu Özkartal, Marco Bergonti, Maria Luce Caputo, Luigi Pannone, Carlo de Asmundis, Paolo Compagnucci, Michela Casella, Paola Berne, Gavino Casu, Federico Migliore, Nicolò Martini, Daniele Faccenda, Esther Scheirlynck, Giulio Conte

**Affiliations:** Cardiology Department, Cardiocentro Ticino Institute, Ente Ospedaliero Cantonale, Via Tesserete 48, Lugano, CH 6900, Switzerland; Faculty of Biomedical Sciences, Università Della Svizzera Italiana, via la Santa 1, Lugano 6962, Switzerland; Cardiology Department, Cardiocentro Ticino Institute, Ente Ospedaliero Cantonale, Via Tesserete 48, Lugano, CH 6900, Switzerland; Faculty of Biomedical Sciences, Università Della Svizzera Italiana, via la Santa 1, Lugano 6962, Switzerland; Cardiology Department, Cardiocentro Ticino Institute, Ente Ospedaliero Cantonale, Via Tesserete 48, Lugano, CH 6900, Switzerland; Faculty of Biomedical Sciences, Università Della Svizzera Italiana, via la Santa 1, Lugano 6962, Switzerland; Heart Rhythm Management Centre, Postgraduate Program in Cardiac Electrophysiology and Pacing, Universitair Ziekenhuis Brussel-Vrije Universiteit Brussel, European Reference Networks Guard-Heart, Brussels, Belgium; Heart Rhythm Management Centre, Postgraduate Program in Cardiac Electrophysiology and Pacing, Universitair Ziekenhuis Brussel-Vrije Universiteit Brussel, European Reference Networks Guard-Heart, Brussels, Belgium; Cardiology and Arrhythmology Clinic, University Hospital ‘Ospedali Riuniti’, Ancona, Italy; Cardiology and Arrhythmology Clinic, University Hospital ‘Ospedali Riuniti’, Ancona, Italy; Department of Cardiology, Ospedale Santissima Annunziata, University of Sassari, Sassari, Italy; Department of Cardiology, Ospedale Santissima Annunziata, University of Sassari, Sassari, Italy; Department of Cardiac, Thoracic and Vascular Sciences and Public Health, University of Padova, Via Giustiniani 2, Padova 35121, Italy; Department of Cardiac, Thoracic and Vascular Sciences and Public Health, University of Padova, Via Giustiniani 2, Padova 35121, Italy; Cardiology Department, Cardiocentro Ticino Institute, Ente Ospedaliero Cantonale, Via Tesserete 48, Lugano, CH 6900, Switzerland; Cardiology Department, Cardiocentro Ticino Institute, Ente Ospedaliero Cantonale, Via Tesserete 48, Lugano, CH 6900, Switzerland; Cardiology Department, Cardiocentro Ticino Institute, Ente Ospedaliero Cantonale, Via Tesserete 48, Lugano, CH 6900, Switzerland; Faculty of Biomedical Sciences, Università Della Svizzera Italiana, via la Santa 1, Lugano 6962, Switzerland

**Keywords:** Brugada syndrome, Bradycardia, Atrioventricular block, Sinus node disease, Sick sinus syndrome, Ventricular arrhythmia

## Introduction

Brugada syndrome (BrS) is an inherited arrhythmia syndrome associated with ventricular arrhythmias (VA) and sudden cardiac death (SCD).^1^ Furthermore, it is associated with sick sinus syndrome (SSS) and atrioventricular conduction disease (AVCD) with reported incidences of 1.1–1.7% and 2.7%, respectively.^2–6^ It was further hypothesized that specific *SCN5A* mutations may be associated with significant bradyarrhythmias.^7^

Whether bradyarrhythmias represent independent phenotypic manifestations or correlate with increased VA risk remains uncertain. This study aims to explore the prognostic significance of bradyarrhythmias in BrS patients.

## Methods and statistical analysis

This retrospective, multicenter study included BrS patients with ECG-documented non-vagal bradycardia, defined as SSS [symptomatic sinus arrest ≥3 s, asymptomatic arrest >6 s, or chronotropic incompetence requiring pacemaker (PM) implantation], AVCD [second degree atrioventricular (AV) block or higher], or both (binodal disease). The study was approved by the Swiss local ethics committee (*BASEC 2025-00524; Rif. CE 4807*), whereas all other centres were responsible for their local approvals.

BrS diagnosis was established according to latest guidelines.^1^ Patients were divided into three groups: isolated SSS, isolated AVCD, and binodal disease.

Primary endpoint was VA occurrence, defined as sustained ventricular tachycardia or ventricular fibrillation. Secondary endpoints were all-cause death and device related complications.

Continuous variables are presented as median (first–third quartile). Categorical variables were compared using Fisher’s exact or Chi-square test, as appropriate. Continuous variables were analysed using the Mann–Whitney U test. Hazard ratios (HRs) with 95% confidence intervals (CIs) were estimated using Cox proportional hazards regression. Given the limited number of events, multivariable modelling was not performed to avoid overfitting. A two-sided *P*-value <0.05 was considered significant. Analyses were performed using GraphPad Prism version 11.0.0 (GraphPad Software, San Diego, CA, USA).

## Results

Among 2308 screened BrS patients, 44 (1.9%) presented documented bradycardia and were included in the study. Baseline characteristics and between-group differences are summarized in Table 1.

**Table 1:** The provided P-values reflect the comparison between isolated AVCD and isolated SSS groups, whereas no between group com parisons were performed for the binodal disease group, given its limited patient and event numbers

Baseline characteristics	Total (*N* = 44)	Binodal disease (*N* = 3)	Isolated AVCD (*N* = 13)	Isolated SSS (*N* = 28)	*P*-value
Age at bradycardia diagnosis, years, median (IQR)	45.9 (36.4–57.7)	69 (32–78)*	50.5 (42.9–56.6)	39 (25.8–50)	0.09
Age at BrS diagnosis, years, median (IQR)	45.5 (37–52.3)	69 (32–86)^a^	49 (45–50)	42 (32–52)	0.17
Male sex, *n* (%)	27 (61.4)	2 (66.7)	8 (61.5)	17 (60.7)	0.96
Proband status, *n* (%)	26 (59.1)	1 (33.3)	7 (53.8)	18 (64.3)	0.52
Family history of BrS, *n* (%)	4/20 (20)	2 (66.7)	2 (15.4)	0/5 (0)	1.0
Family history of SCD, *n* (%)	7 (15.9)	1 (33.3)	1 (7.7)	5 (17.9)	0.64
History of syncope, *n* (%)	29 (65.9)	3 (100)	9 (69.2)	17 (60.7)	0.73
Vagal, *n* (%)	6 (20.7)	1 (33.3)	4 (30.8)	1 (5.9)	
Arrhythmic, *n* (%)	6 (20.7)	1 (33.3)	3 (23.1)	2 (11.8)	
Undetermined, *n* (%)	17 (58.6)	1 (33.3)	6 (46.2)	14 (82.4)	
Spontaneous Brugada type 1 ECG	17 (38.6)	2 (66.7)	6 (46.2)	9 (32.1)	0.39
1st degree AV block	19 (43.2)	2 (66.7)	6 (46.2)	11 (39.3)	0.68
PR interval, median (IQR), ms	191 (158–218)	230 (185–305)*	200 (160–212)	181 (151–215)	0.54
QRS interval, median (IQR), ms	100 (90–118)	110 (105–143)*	100 (90–104)	102 (91–120)	0.47
Right bundle branch block	7 (15.9)	0 (0)	2 (15.4)	5 (17.9)	1.0
Genetic test performed, *n* (%)	34 (77.3)	2 (66.7)	6 (46.2)	26 (92.9)	<0.001
*SCN5A* P/LP variant	14 (41.2)	2 (100)	0 (0)	12 (46.2)	0.06
CIED	36 (81.8)	3 (100)	9 (69.2)	24 (85.7)	
Defibrillator, *n* (%)	30 (68.2)	2 (66.7)	5 (38.5)	23 (82.1)	0.005
Pacemaker, *n* (%)	6 (13.6)	1 (33.3)	4 (30.8)	1 (3.6)	0.028
AP, %, mean (min-max)	5 (0–47)	47.5 (6–89)	0.7 (0–7.5)	18.8 (0–54.5)	0.24
VP, %, mean (min-max)	1 (0–38)	4.5 (1–8)	3.1 (1–28.5)	1 (0–42)	0.32
Device related complication, *n* (%)	9 (20.5)	1 (33.3)	0 (0)	8 (28.6)	0.04
Lead malfunction, *n* (%)	7 (77.8)	1 (100)	0 (0)	6 (75)	
Inappropriate shock, *n* (%)	1 (11.1)	0 (0)	0 (0)	1 (12.5)	
Device infection, *n* (%)	1 (11.1)	0(0)	0 (0)	1 (12.5)	
Clinical outcome					
Follow-up, years, median (IQR)	6.7 (3.6–10.2)	8.6 (7.0–12.4)*	6.0 (3.4–7.4)	7.0 (4.2–12.3)	0.13
Ventricular arrhythmias, *n* (%)	11 (25.0)	1 (33.3)	2 (15.4)	8 (28.6)	0.46
Ventricular fibrillation, *n* (%)	7 (63.6)	1 (100)	1 (50)	5 (62.5)	
Ventricular tachycardia, *n* (%)	4 (36.4)	0 (0)	1 (50)	3 (37.5)	
Death, *n* (%)	5 (11.4)	1 (33.3)	0 (0)	4 (14.3)	0.28
Non-cardiovascular, *n* (%)	3 (60)	1 (100)	0 (0)	2 (50)	
Unknown cause, *n* (%)	2 (40)	0 (0)	0 (0)	2 (50)	

AP, atrial pacing; AV, atrio-ventricular; AVCD, atrio-ventricular conduction diesase; BrS, Brugada syndrome; CIED, cardiac implantable cardioverter defibrillator; IQR, inter quartile range; SCD, sudden cardiac death; *SCN5A*, sodium channel protein type 5 subunit alpha; SSS, sick sinus syndrome; VP, ventricular pacing.

^a^In these patients, the stated numbers are median (minimum-maximum).

Isolated AVCD was present in 13 (29.5%), SSS in 28 (63.6%), and binodal disease in 3 (6.8%) patients. Among AVCD patients, five had complete AV block, whereas the remainder had second degree or advanced block, mainly paroxysmal (88% of cases). In nine patients, bradycardia preceded BrS diagnosis (four AVCD and five SSS), by a median of 3.6 years (interquartile range 0.5–6.8).

History of syncope was present in 66% of patients, with vagal and arrhythmic causes accounting for 21% each. Genetic testing was performed in 77.3% overall, significantly more often in SSS than AVCD (92.9% vs. 46.2%, *P* < 0.001). *SCN5A* P/LP variants were present in 46.2% of tested SSS (12/26) and absent in tested AVCD patients (0/6). Among patients with binodal disease, two underwent genetic testing, both of which carried *SCN5A* P/LP variants.

Overall, 68% of patients had an implantable cardioverter-defibrillator (ICD) and 14% a PM. ICD was significantly more frequent in the SSS group (82.1 vs. 38.5, *P* = 0.005), whereas PM was more frequent in AVCD patients (30.8 vs. 3.6%, *P* = 0.028). In 6 PM patients, five (83.3%) were implanted due to AVCD, two (33.3%) prior to BrS diagnosis, and only one (16.7%) had VA during follow-up; since he was 87 years of age and refused any intervention, none of the PM patients were upgraded to ICD.

Over a median follow-up of 6.7 years (3.6–10.2), 11 of 44 patients (25%) had VA events: 2/13 in AVCD, 8/28 in SSS, and 1/3 in binodal disease, corresponding to an annual VA rate of 4.0 (95% CI 2.0–7.1) per 100 person-years without between-group differences (*P* = 0.56). Event rates were 4.1 (95% CI 1.8–8.1), 3.0 (95% CI 0.4–10.8), and 6.2 (95% CI 0.2–34.4) per 100 patient-years for SSS, AVCD, and binodal dysfunction, respectively.

Patients experiencing VA were in median 40 years (29–50) old, predominantly male (81.8%) with spontaneous type 1 ECG in 64%, and history of syncope in 91% of cases. Genetic testing was available in 82% of patients showing *SCN5A* P/PL variants in 44.4% of cases. Most patients with VA were ICD carriers (91%), whereas only one had a PM.

Overall mortality was 11.4% (*n* = 5), including one in the binodal and four in the SSS group. Two deaths were of unknown cause, while the remaining were non-cardiovascular.

Device-related complications occurred in 20.5% of patients, corresponding to an event rate of 3.4 (95% CI 1.5–6.7) per 100 patient-years, with lead malfunction requiring revision being the most frequent (77.8%). All complications occurred in ICD patients, and none in PM patients (30% vs. 0%, *P* = 0.30).

## Discussion and conclusion

This study describes a cohort of BrS patients with bradycardia, comparing SSS, AVCD, and binodal disease phenotypes. The prevalence of documented bradycardia was low (1.9%). The overall VA incidence was four events per 100 person-years, which is higher than previously reported rates of approximately 1.5% per year.^8,9^

VA incidence was numerically higher in SSS compared with AVCD patients, but the difference did not reach statistical significance.

Device-related complications were common with a cumulative incidence of 20.5%, mainly driven by ICD lead issues.

The relatively high rate of SCN5A variants in SSS patients supports the concept that sodium-channel alterations may affect electrical impulse generation, conduction, and ventricular arrhythmogenesis, reinforcing the hypothesis of BrS being a heterogeneous electrical disease extending beyond ventricular manifestations.^10^ However, whether bradyarrhythmia represents an independent risk factor or a marker of advanced electrophysiological disease remains unclear.

Our findings should be considered hypothesis generating in light of some limitations. This is a retrospective study bearing inherent limitations related to its design. Furthermore, due to phenotype rarity, the sample size is limited and the results must be interpreted with caution. Finally, given the lower testing rate in AVCD, ascertainment bias cannot be completely excluded. Larger multicenter cohort studies are necessary to better define the clinical implications and risk stratification of BrS patients with concomitant bradycardia.

In the meantime, our findings suggest that bradyarrhythmia in BrS patients should prompt thorough arrhythmic risk stratification and careful device selection, given the non-negligible rate of device-related complications.

## Data Availability

Data is available upon reasonable request and with the consent of all co-authors.
